# The pediatric Cogni-Famille protocol: family-mediated manual therapy achieves comparable outcomes at one-eighth cost in children with cerebral palsy—a franco-Cameroonian comparative study

**DOI:** 10.3389/fped.2026.1797591

**Published:** 2026-08-04

**Authors:** Ibrahim Npochinto Moumeni, Njikam Moumeni Abdel-Nasser

**Affiliations:** 1 Department of Physical Therapy & Physical Medicine, Faculty of Medicine and Pharmaceutical Sciences, University of Dschang, Dschang, Cameroon; 2 Department of Physical Medicine & Osteopathy, Regional Hospital of Bafoussam, Bafoussam, Cameroon; 3 Institute for Applied Neurosciences and Functional Rehabilitation (INAREF), Odza-Yaoundé, Cameroon; 4 Franco-African Center for Applied Rehabilitation and Health Sciences (CFARASS), Foumbot, Cameroon; 5 Department of Geriatrics and Gerontology, Sorbonne Université, Pitié-Salpêtrière Hospital, Paris, France; 6 Private Practitioner, Paris, France; 7 Faculty of Health Sciences, University of Parakou, Parakou, Benin; 8 French-Speaking African Society for Neurorehabilitation(SAFNeR), Parakou, Benin; 9 UREKIM—Research Unit in Physiotherapy and Physical Medicine, Faculty of Medicine and Pharmaceutical Sciences, University of Dschang, Dschang, Cameroon; 10 Centre de Recherche en Santé Humaine et Développement des Médicaments (CRESHDEM), Faculty of Medicine and Pharmaceutical Sciences, University of Dschang, Dschang, Cameroon

**Keywords:** Cameroon, cerebral palsy, Cogni-Famille protocol, contextual rehabilitation, cost comparison, family-mediated therapy, low-resource settings, stigmatization

## Abstract

**Background:**

Cerebral palsy (CP) remains the leading childhood motor disorder worldwide, yet rehabilitation access is severely limited across sub-Saharan Africa due to resource scarcity, workforce shortages, and profound cultural stigmatization. Family-mediated approaches offer a promising pathway to bridge this gap. To our knowledge, this study represents the first evaluation of a pediatric adaptation of the Cogni-Famille protocol — originally developed for adult post-stroke rehabilitation — applied to children with spastic CP across two dramatically different resource environments.

**Methods:**

Twenty-one children with confirmed spastic CP (GMFCS levels I–III, ages 5–12) completed a 12-week family-mediated manual therapy (FMMT) intervention: 9 in institutional settings (France) and 12 in home-based care (Cameroon). Caregivers received progressive training in five manual therapy modules emphasizing ecological plasticity principles, delivered through twice-weekly therapist-supervised sessions (45–60 min each) complemented by daily home practice of 2–3 h. Primary outcome was change in Gross Motor Function Measure (GMFM-66). Secondary outcomes included spasticity (Modified Ashworth Scale), exploratory cost comparison, and qualitative social impact assessment. Statistical comparisons used Mann–Whitney U tests for continuous variables and Fisher's exact test for categorical variables.

**Results:**

Both cohorts achieved clinically significant GMFM-66 improvements (France: +15.2 ± 3.8 points; Cameroon: +13.8 ± 4.2 points; *p* = 0.28), exceeding the minimal clinically important difference by 8-fold. Spasticity decreased comparably across settings (Modified Ashworth: France −1.3 ± 0.4; Cameroon −1.1 ± 0.5; *p* = 0.33). Exploratory cost comparison revealed the Cameroonian model cost one-eighth that of France (38€ vs. 320€ monthly) while achieving equivalent clinical outcomes. Qualitative interviews documented profound social impacts: 11 of 12 Cameroonian families reported reduced stigmatization and improved community integration. Three Cameroonian children died during follow-up from complications unrelated to the intervention, reflecting the significant health vulnerabilities faced by children with disabilities in resource-limited settings.

**Conclusions:**

Family-mediated manual therapy produces substantive motor improvements at dramatically reduced cost when culturally contextualized, while simultaneously addressing social exclusion. This approach demonstrates five innovative mechanisms — ecological plasticity, therapeutic motherhood, sequential micro-learning, community anchoring, and functional dignity restoration — offering a scalable and replicable model for addressing Africa's rehabilitation gap.

## 1 Introduction

Cerebral palsy (CP) remains the most common childhood motor disorder worldwide, with global prevalence estimated between 1.5 and 4 per 1,000 live births (1, 2). While contemporary approaches to motor rehabilitation have advanced significantly in high-resource settings, access to specialized services remains severely limited across much of Africa, where prevalence may be considerably higher due to limited perinatal care (3, 4). Beyond clinical considerations, children with CP throughout sub-Saharan Africa face profound social exclusion rooted in cultural beliefs that fundamentally alter their life trajectory.

In many West and Central African communities, including Cameroon, children with CP face profound social exclusion rooted in cultural beliefs that fundamentally alter their life trajectory. Commonly described as “snake children” (enfants-serpents) or “cursed infants” (enfants maudits), these children are subjected to stigmatization that extends beyond the individual to encompass their families, especially mothers who are often blamed for the child's condition (5–7). The consequences are severe: fathers frequently abandon these families, children remain hidden at home, and access to education and social interaction is denied (8, 9). This stigmatization compounds practical challenges in the rehabilitation landscape, where formal pediatric services remain dramatically insufficient, with significant urban-rural disparities (10–12). According to Moumeni et al. (13), many rural Cameroonian regions have only one rehabilitation specialist per 200,000 inhabitants (59), creating effective “rehabilitation deserts” where therapeutic opportunities are essentially nonexistent.

Against this challenging backdrop, recent research has demonstrated the effectiveness of family-centered approaches in high-resource settings (14–16). Task-oriented, intensive, and repetitive interventions delivered within meaningful contexts can effectively leverage neuroplasticity principles to improve motor outcomes (17–19). The original Cogni-Famille protocol was developed for adult stroke patients, transferring key therapeutic techniques from professionals to trained family members, showing promising results in promoting both neuroplasticity and functional independence (20, 21). To our knowledge, this study represents the first systematic evaluation of the Cogni-Famille protocol adapted for children with cerebral palsy. While the original protocol was developed for adult post-stroke rehabilitation, its core principles — neuroplasticity activation through intensive repetition, task-shifting to trained family members, and ecological continuity of therapeutic stimulation — are directly transposable to pediatric motor development. The adaptation required contextual modifications: play-based task framing, shorter session durations calibrated to children's attentional capacity, and caregiver training emphasizing developmental sequencing rather than stroke recovery. The theoretical basis for this transposition rests on shared neuroplasticity mechanisms between post-lesional recovery and developmental motor learning.

This pediatric adaptation explores whether similar principles can be successfully implemented in a culturally contextualized form for children with CP across dramatically different resource environments. Specifically, we aimed to determine: (1) whether family-mediated manual therapy (FMMT) could yield comparable motor outcomes across institutional (France) and home-based (Cameroon) settings; (2) what economic differential exists between these contexts; and (3) what social impacts might extend beyond motor function.

We hypothesized that active parental involvement and cultural contextualization would enable comparable functional gains across settings, potentially addressing both motor and social dimensions of childhood disability. By creating a replicable, low-cost intervention model specifically adapted to resource-limited settings, this study not only tests principles of neuroplasticity but also examines how therapeutic approaches can be contextually reimagined to combat stigmatization and promote social inclusion. Through this bi-continental comparative approach, we seek to provide evidence-based solutions to the substantial rehabilitation gap facing millions of children with CP across Africa and other resource-constrained environments.

## 2 Methods

### 2.1 Study design, ethics, and regulatory compliance

This investigation employed a retrospective comparative cohort design spanning from January 2020 through March 2024, with data collection conducted between 2020 and 2022 and follow-up continuing through 2023–2025. All procedures were conducted in full compliance with international and national ethical standards for human subjects research.

For the French cohort, this study adhered to the regulatory framework established by the French Public Health Code, specifically Articles L.1121-1 and R.1121-2, which stipulate that non-interventional retrospective studies using anonymized clinical data do not require formal ethics committee approval but must comply with data protection regulations. Data processing was conducted in accordance with the Methodology Reference MR-004 of the Commission Nationale de l'Informatique et des Libertés (CNIL), which governs retrospective analysis of anonymized healthcare data. All participating families provided informed consent for their anonymized data to be used for research purposes.

For the Cameroonian cohort, the study was conducted under Institutional Certification N°43/DRSO/HRB/55/2023 from the Regional Hospital of Bafoussam, Department of Physical and Rehabilitation Medicine & Medical Osteopathy. As certified by Dr. ALIMA Jean Marie, Deputy Director, “retrospective clinical evaluations based exclusively on anonymized data obtained from routine patient care do not require prior approval by an ethics committee, provided they are conducted under the direct coordination and oversight of the responsible department chief.” The certification confirms that these observational reviews were “strictly non-interventional, involve no experimental procedures, no deviation from standard medical protocols, and maintain full compliance with confidentiality standards and patient rights in line with both national guidelines and international ethical principles, including those outlined in the Declaration of Helsinki.”

All data were anonymized and analyzed in accordance with the principles of the Declaration of Helsinki. Informed consent was obtained from all participating families, with particular care taken to ensure comprehension among Cameroonian participants with varying literacy levels through verbal explanation in local languages (primarily Bamoun and Bamiléké) with culturally appropriate documentation procedures.

### 2.2 Participants

Children were eligible if they had a confirmed diagnosis of spastic cerebral palsy (GMFCS levels I–III), were aged 5–12 years, maintained stable medication throughout the study period, and had not undergone orthopedic surgery in the preceding six months. Exclusion criteria included severe cognitive impairment that would prevent comprehension of play-based tasks, uncontrolled seizure disorders, or medical instability.

Given the retrospective observational design and the absence of prior data on family-mediated manual therapy in pediatric CP across bi-continental settings, formal *a priori* sample size calculation was not feasible. The sample size reflects the total number of eligible children meeting inclusion criteria followed in the two clinical settings during the study period. This pragmatic sampling approach is consistent with retrospective case series methodology and is acknowledged as a limitation of the study design.

A total of 24 children were Initially enrolled (10 In France, 14 In Cameroon). During the study period, three children in the Cameroonian cohort died: two during the first year of the study (one from cerebral malaria complications and one from respiratory infection) and one during the second year (from respiratory complications following influenza). These deaths were not related to the intervention but reflect the significant health vulnerabilities faced by children with disabilities in resource-limited settings. Of the remaining 21 children, all completed the full intervention protocol: 9 in France and 12 in Cameroon. Baseline demographic and clinical characteristics are presented in Table 1.

**Table 1:** Baseline demographic and clinical characteristics of participants.

Characteristic	France (*n* = 9)	Cameroon (*n* = 12)	*p*-value
Demographics			
Age (years), mean ± SD	8.4 ± 2.1	7.7 ± 2.5	0.47
Sex (male/female), *n*	5/4	7/5	0.58
GMFCS level, *n* (%)			
Level I	3 (33.3%)	4 (33.3%)	0.66
Level II	4 (44.4%)	6 (50.0%)	0.53
Level III	2 (22.2%)	2 (16.7%)	0.57
Clinical Parameters			
Baseline GMFM-66, mean ± SD	54.6 ± 8.7	52.9 ± 7.9	0.63
Baseline Modified Ashworth Scale, mean ± SD	2.8 ± 0.6	2.9 ± 0.7	0.71
Socioeconomic Context			
Monthly household income (€), mean ± SD	1,680 ± 420	72 ± 28	<0.001*
Previous rehabilitation access, *n* (%)	9 (100%)	3 (25%)	<0.001*
Primary caregiver (mother/grandmother), *n*	9/0	9/3	0.04*
Paternal legal recognition, *n* (%)	9 (100%)	4 (33.3%)	<0.001*

Baseline demographic and clinical characteristics of participants in the Cogni-Famille Pediatric Adaptation study. Despite comparable clinical parameters (age, GMFCS distribution, baseline GMFM-66, and Modified Ashworth Scale scores), profound socioeconomic disparities exist between cohorts. Cameroonian families have dramatically lower household income (23-fold difference), severely limited previous rehabilitation access (75% had never received rehabilitation), and compromised family structures with only one-third of children legally recognized by fathers. Three Cameroonian children (25%) were cared for by grandmothers after maternal abandonment. These contextual differences underscore the intervention's effectiveness across dramatically different resource environments. GMFCS: Gross Motor Function Classification System; GMFM-66: Gross Motor Function Measure-66; SD: Standard Deviation.

* Statistically significant (*p* < 0.05).

### 2.3 Recruitment strategy and cultural adaptations

The significant contextual differences between sites necessitated distinct recruitment approaches. In France, participants were referred through established medical channels and institutional rehabilitation networks. All French participants received care in institutional settings with trained therapists supervising family members.

In Cameroon, a specialized outreach strategy was developed to address the profound social isolation experienced by target families. Children were primarily identified through an incidental identification approach during general pediatric consultations for common illnesses (malaria, gastrointestinal infections, malnutrition) rather than for their motor disability. This approach provided access to children who were typically kept at home due to social stigma.

Families were carefully informed that rehabilitation would occur at home, respecting cultural sensitivities and privacy concerns. This approach minimized community visibility and associated stigma while providing intervention in a familiar environment. Cost was limited to therapist transportation expenses (approximately 25,000 FCFA or 38€ monthly), as most families survived on subsistence agriculture.

Cultural adaptations included: (1) provision of pictorial handouts tailored to literacy level with simplified text in appropriate languages (French, Bamoun, and Bamiléké), (2) extensive demonstration of techniques rather than written instructions, (3) adaptation of all materials to use objects readily available in rural Cameroonian households, requiring no specialized equipment, and (4) therapist travel to each family's home rather than requiring families to attend a clinical facility.

### 2.4 Intervention protocol: cogni-famille pediatric adaptation

The intervention systematically adapted the adult Cogni-Famille methodology (20, 21) for pediatric application by incorporating developmental principles and play-based elements. The transposition rests on shared neuroplasticity mechanisms between post-lesional recovery and developmental motor learning (22–24): intensive repetition within ecologically meaningful contexts drives functional neural reorganisation regardless of whether the underlying pathology involves stroke or congenital motor impairment. Three contextual modifications distinguished the paediatric version from the original protocol: play-based task framing, session durations calibrated to children's attentional capacity, and caregiver training structured around developmental sequencing rather than stroke-recovery milestones. The core approach remained consistent across both sites, although delivery contexts differed substantially — institutional settings in France vs. home-based delivery in Cameroon.

#### 2.4.1 Sequential modular structure

The programme comprised five sequential modules taught progressively to one primary family member (typically the mother or grandmother). Consistent with sequential micro-learning principles (25, 26), each module was introduced only after demonstrated mastery of the previous one, preventing cognitive overload while building caregiver confidence. Mastery was defined as correct technique execution on three consecutive supervised occasions. The five modules, their objectives, core techniques, implementation parameters, and required materials are summarised in Table 2.

**Table 2:** Summary of the five sequential modules of the cogni-famille pediatric adaptation protocol.

Module (Weeks)	Objective	Core techniques	Implementation	Materials
1 — Dynamic Stretching (Wks 1–3)	Reduce spasticity; prevent contractures	Slow sustained elongation of spastic muscle groups (hamstrings, gastrocnemius, hip adductors, forearm flexors); progressive passive-to-active transitions; child-appropriate distraction	10–15 min, twice daily	Blankets, pillows (household positioning aids)
2 — Guided Amplitude Movements (Wks 3–5)	Increase functional range of motion and movement control	Controlled movement through available range; progressive weight-bearing with weight-shifting; functional positioning for daily activities	15–20 min, twice daily; integrated with routine activities	Water bottles, sticks, chairs
3 — Functional Play (Wks 5–7)	Develop goal-directed movements within meaningful activities	Grasp-release training through age-appropriate games; throwing/catching with progressive complexity; task-specific practice (self-feeding, dressing)	20–30 min daily in playful sessions	Locally available toys, household items, culturally relevant play materials
4 — Imitation & Coordination (Wks 7–9)	Enhance motor planning, bilateral coordination and sequencing	Symbolic gesture imitation with increasing complexity; rhythm-based coordination (clapping, drumming); bilateral integration tasks (object transfer, two-handed tasks)	15–20 min, twice daily	Drums, rattles, or improvised musical alternatives
5 — Postural Control & Breathing (Wks 9–12)	Improve core stability, postural transitions, and respiratory coordination	Core activation with respiratory coordination; midline orientation and crossing activities; transitional movement sequences for functional mobility	Integrated throughout daily routines	Floor mats or blankets, supportive seating

Wks, weeks; min, minutes.

#### 2.4.2 Implementation framework

The standardised framework ensured consistency while allowing necessary cultural and contextual adaptations. Two supervised therapist visits were conducted weekly (45–60 min each), complemented by family-led home practice twice daily (15–20 min per session). Weekly progress evaluations guided programme adjustments. In France, sessions were conducted by certified physiotherapists in institutional settings. In Cameroon, the principal investigator and a trained physiotherapy collaborator delivered all sessions at each family's home. Pictorial handouts tailored to literacy level were provided in French and local languages (Bamoun and Bamiléké), with extensive technique demonstration prioritised over written instruction. All Cameroonian materials used objects readily available in rural households, requiring no specialised equipment. Caregiver adherence exceeded 85% in both cohorts across the 12-week training phase, documented through weekly session logs completed by the supervising therapist during each home visit. Independent verification of between-session home practice was not feasible given the retrospective design and resource constraints of community-based delivery; this constitutes a methodological limitation, and future prospective studies should incorporate objective adherence measurement (caregiver-completed activity diaries, video verification, or wearable sensors).

#### 2.4.3 Quality control and conceptual framework

Several quality control measures maintained intervention fidelity across sites: standardised therapist training protocols, video review of sample sessions to verify technique consistency, regular case discussions between the French and Cameroonian clinical teams, structured observation checklists for family-member proficiency, and graduated skill assessment before module progression.

**Figure 1:**
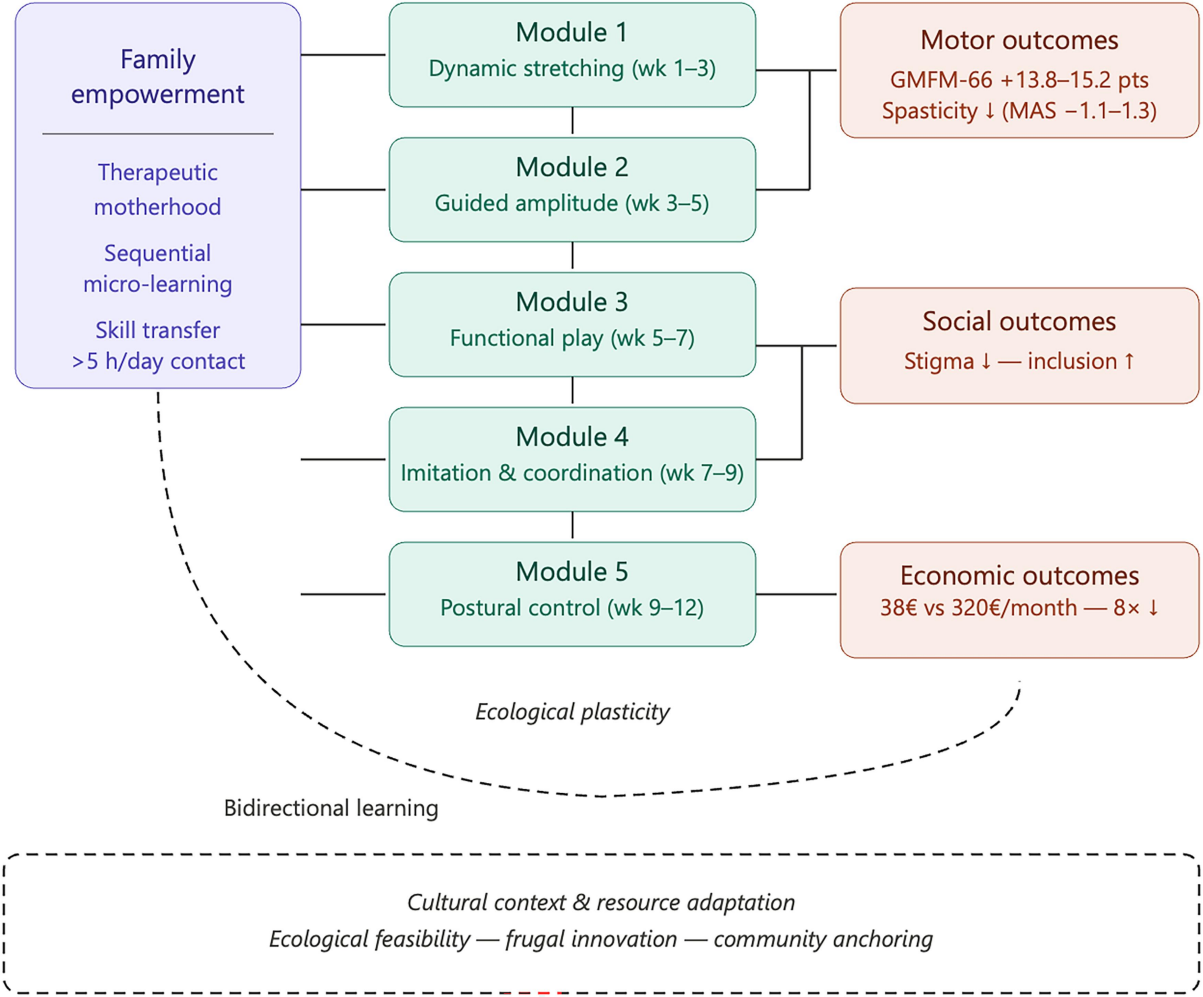
Conceptual framework of the Cogni-Famille Pediatric Adaptation. Family empowerment through therapeutic motherhood and systematic skill transfer enables delivery of five sequential modules (dynamic stretching, guided amplitude movements, functional play, imitation and coordination, and postural control with breathing). These modules, embedded within a foundational layer of cultural context and resource adaptation (dotted box), generate outcomes across four domains: motor improvement (GMFM-66), spasticity reduction (Modified Ashworth Scale), social inclusion, and stigma reduction. Solid arrows indicate directional influences; bidirectional arrows represent the reciprocal relationship between caregiver skill acquisition and module delivery, wherein functional gains reinforce caregiver confidence in a positive feedback loop. The feedback arc at the base illustrates cross-contextual knowledge transfer between high- and low-resource implementation settings. GMFM-66: Gross Motor Function Measure-66; MAS: Modified Ashworth Scale.

The conceptual logic underlying these measures is captured in Figure 1, which illustrates how family empowerment through therapeutic motherhood and systematic skill transfer enables delivery of the five sequential modules. Each module, embedded within cultural context and resource adaptation, generates outcomes across four domains: motor improvement (GMFM-66), spasticity reduction (Modified Ashworth Scale), social inclusion, and stigma reduction. The bidirectional arrows in the figure represent the reciprocal relationship between caregiver skill acquisition and module delivery: functional gains achieved through each module reinforce caregiver motivation and confidence in a positive feedback loop (27, 28). Solid arrows indicate directional influences; the feedback arc at the base illustrates bidirectional knowledge transfer between the two implementation contexts, reflecting the cross-contextual learning described in Section 4. The dotted box represents the foundational layer of cultural context and resource adaptation that enables feasibility and sustainability across settings (24, 29, 30).

### 2.5 Outcome measures

Primary outcome: Change in Gross Motor Function Measure (GMFM-66) from baseline to program completion at 12 weeks. This validated tool provides a standardized assessment of gross motor function in children with CP, with established reliability and validity across cultural contexts (31).

**Secondary outcomes included:**

- Modified Ashworth Scale (MAS) to assess changes in spasticity
- Family satisfaction questionnaire (0–10 scale)
- Economic impact assessment (direct and indirect costs)
- Qualitative interviews on social participation and stigma experience

Assessments were conducted at baseline, 6 weeks, and 12 weeks by trained evaluators blind to study hypotheses but not to group assignment due to obvious contextual differences. In Cameroon, additional 6-month follow-up data were collected when possible (8/12 participants). In the French cohort, GMFM-66 assessments were conducted by the supervising physiotherapist who also delivered the training sessions. In the Cameroonian cohort, both intervention delivery and outcome assessment were performed by the principal investigator. The risk of assessor bias inherent to this dual role is acknowledged as a limitation. To mitigate this, standardized GMFM-66 administration protocols were strictly followed at every assessment point, with scoring performed according to validated CanChild criteria. Future prospective studies should employ independent assessors blinded to intervention delivery.

### 2.6 Statistical analysis

Sample size was determined by available eligible participants meeting inclusion criteria during the study period rather than *a priori* power calculations. Descriptive statistics were calculated for all variables (means, standard deviations, frequencies, and percentages). Comparative analyses between sites used independent *t*-tests for continuous variables and chi-square or Fisher's exact test for categorical data. Paired *t*-tests assessed within-group changes from baseline to post-intervention. Statistical significance was set at *p* < 0.05. All analyses were performed using SPSS version 26 (IBM Corp., Armonk, NY).

Qualitative data from family interviews were analyzed using thematic content analysis with open coding by two independent researchers. Initial codes were grouped into themes and subthemes, with discrepancies resolved through consensus discussion. Trustworthiness was enhanced through member checking with a subset of participants.

## 3 Results

### 3.1 Socio-demographic characteristics and contextual differences

Initial family interviews in the Cameroonian cohort revealed distressing social circumstances that were not present in the French cohort. Eight of twelve Cameroonian children (66.7%) lacked legal recognition from their fathers, who had abandoned the family after the child's disability became apparent. Three mothers (25%) had subsequently abandoned their children to grandmothers, who became the primary caregivers despite advanced age and extreme poverty.

Qualitative interviews documented severe social exclusion, with families reporting derogatory terminology used by community members, including “enfant-serpent” (snake-child), “survivant” (survivor, implying others like them should have died), and “famille maudite” (cursed family). These stigmatizing attitudes directly impacted healthcare-seeking behaviors, with many families reporting previous attempts to access traditional healers or religious cures before considering rehabilitation.

The economic contexts differed dramatically between sites. French families had a mean monthly income of 1,680 ± 420€, with all children having received previous rehabilitation services. In contrast, Cameroonian families had a mean monthly income of 72 ± 28€, with only 3/12 children (25%) having previously accessed any form of rehabilitation services. Even the modest sum required for therapist transportation (38€ monthly) represented a substantial financial commitment for participating Cameroonian families.

#### 3.1.1 Participant flow and adherence

**Figure 2:**
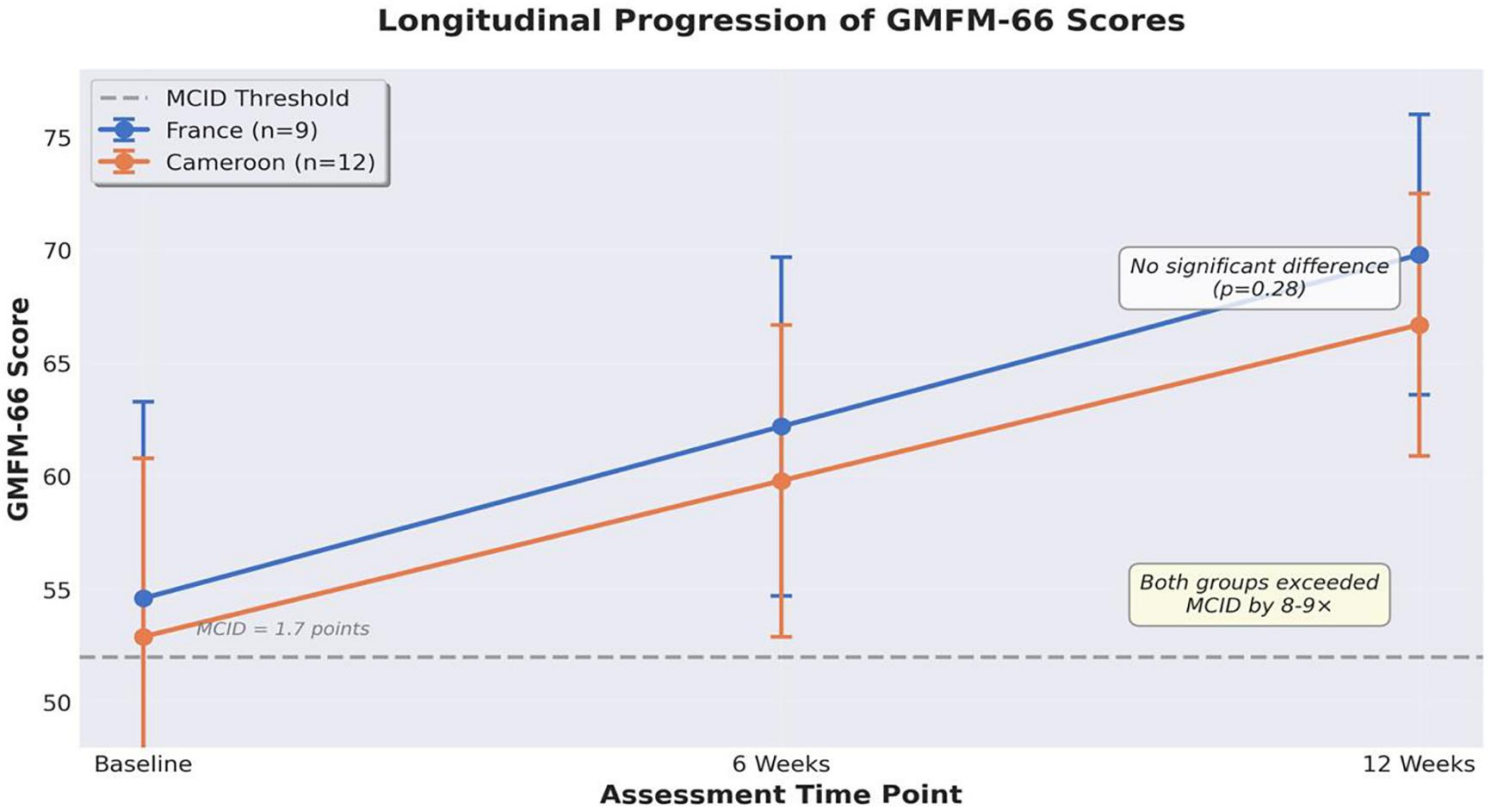
Longitudinal progression of Gross Motor Function Measure-66 (GMFM-66) scores comparing institutional rehabilitation in France (*n* = 9, blue line) and home-based care in Cameroon (*n* = 12, orange line) across three assessment time points: baseline, 6 weeks, and 12 weeks. Both cohorts demonstrated parallel improvement patterns with no statistically significant between-group differences at any time point. France progressed from baseline 54.6 ± 8.7 to 69.8 ± 6.2 points [+15.2 ± 3.8; 95% CI (+12.3, +18.1); *p* < 0.001 within-group], while Cameroon advanced from 52.9 ± 7.9 to 66.7 ± 5.8 points [+13.8 ± 4.2; 95% CI (+11.1, +16.5); *p* < 0.001 within-group]. No statistically significant difference was observed between cohorts at any time point (12-week comparison: *p* = 0.28). Formal equivalence testing was not performed given the exploratory and small-sample nature of this study; the absence of detected differences should not be interpreted as proof of equivalence. The magnitude of improvement in both settings substantially exceeded the minimal clinically important difference (MCID) threshold of 1.7 points (indicated by gray dashed line) by approximately 8–9 times, demonstrating robust clinical meaningfulness despite dramatically different resource contexts (320€ vs. 38€ monthly costs). Error bars represent standard deviations. Mid-point values at 6 weeks were estimated through linear interpolation. The parallel trajectories are consistent with the hypothesis that family-mediated manual therapy can achieve clinically meaningful motor gains across institutional and home-based settings when appropriately contextualized for local cultural and resource realities.

Of the 24 initially enrolled participants (10 in France, 14 in Cameroon), three Cameroonian children died during the study period due to complications unrelated to the intervention (two from infectious diseases in year one and one from respiratory complications following influenza in year two). Among the 21 surviving participants, 18 completed the full intervention protocol, corresponding to an overall retention rate of 85.7% among survivors (75% when calculated from initial enrollment). One French participant withdrew due to medical complications unrelated to the intervention, and two Cameroonian families relocated outside the study area. Adherence to the prescribed home program was high across both sites (France: 92.3% of prescribed sessions; Cameroon: 89.7%) see Figure 2 and Table 3.

**Table 3:** Functional outcomes after 12 weeks of intervention.

Outcome Measure	France (*n* = 9)	Cameroon (*n* = 12)	*p*-value
Primary Outcome: GMFM-66			
Baseline score, mean ± SD	54.6 ± 8.7	52.9 ± 7.9	0.63
Post-intervention score (12 weeks), mean ± SD	69.8 ± 6.2	66.7 ± 5.8	0.28
Change from baseline, mean ± SD	**+15.2 ± 3.8**	**+13.8 ± 4.2**	**0.28**
95% Confidence Interval	[+12.3, +18.1]	[+11.1, +16.5]	—
Within-group *p*-value (paired t-test)	<0.001*	<0.001*	—
Effect size (Cohen's d)^†^	2.01	1.89	—
Times MCID threshold (1.7 points)	8.9×	8.1×	—
Secondary Outcomes			
Modified Ashworth Scale			
Baseline, mean ± SD	2.8 ± 0.6	2.9 ± 0.7	0.71
Post-intervention, mean ± SD	1.5 ± 0.5	1.8 ± 0.6	0.29
Change from baseline, mean ± SD	**−1.3 ± 0.4**	**−1.1 ± 0.5**	**0.33**
Within-group *p*-value	<0.001*	<0.001*	—
Program adherence (% sessions completed), mean ± SD	92.3 ± 6.1	89.7 ± 7.8	0.41
Family satisfaction (0–10 scale), mean ± SD	9.1 ± 0.6	9.4 ± 0.5	0.42

Functional outcomes after 12 weeks of family-mediated manual therapy intervention. Both cohorts demonstrated clinically significant improvements with no statistically significant between-group differences (*p* = 0.28 for GMFM-66; *p* = 0.33 for MAS) in motor function and spasticity reduction. Formal equivalence testing was not performed given the exploratory nature and small sample size of this study; the absence of significant differences should not be interpreted as proof of equivalence but as absence of detected difference. Within-group improvements were highly significant (*p* < 0.001) for both primary and secondary outcomes. Effect sizes exceeded 1.8 in both cohorts, indicating very large treatment effects according to Cohen's criteria (d > 0.8 = large). The observed GMFM-66 improvements were 8–9 times the minimal clinically important difference (MCID) threshold of 1.7 points, demonstrating substantial clinical meaningfulness despite modest sample size. High program adherence (>89%) and family satisfaction (>9/10) were observed across both settings, supporting intervention feasibility and acceptability.

GMFM-66: Gross Motor Function Measure-66; MCID: Minimal Clinically Important Difference; SD: Standard Deviation.

* Statistically significant (*p* < 0.05).

† Effect sizes calculated using Cohen's d formula with pooled standard deviation.

### 3.2 Functional outcomes

Both cohorts demonstrated clinically significant improvements in motor function following the 12-week intervention (Figure 2, Table 2). The magnitude of improvement did not significantly differ between settings despite dramatic differences in resource availability and delivery context.

**Figure 3:**
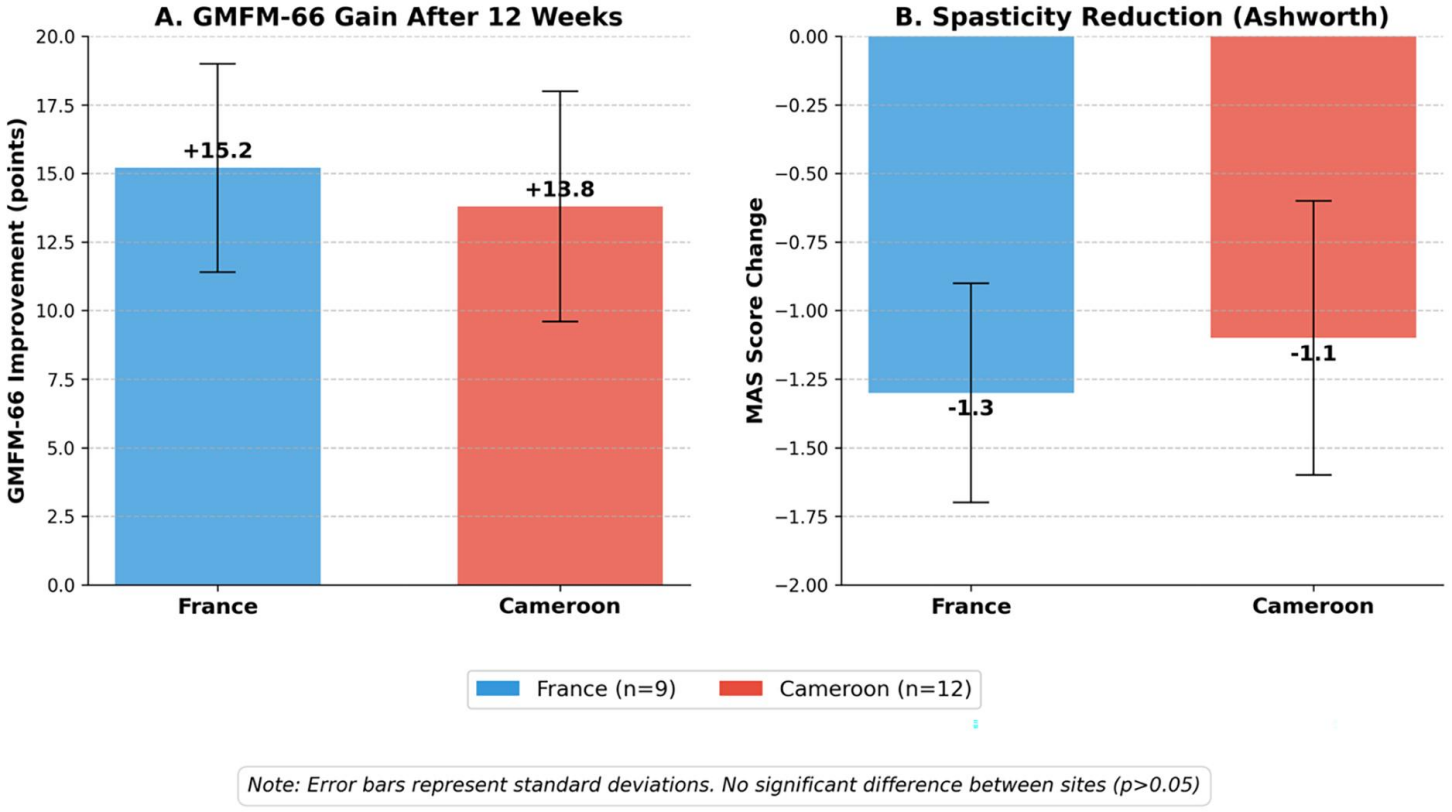
Comparative functional outcomes between participants in France (*n* = 9, blue bars) and Cameroon (*n* = 12, orange bars) following 12 weeks of the Cogni-Famille Pediatric Adaptation intervention. Panel **(A)** displays GMFM-66 improvements from baseline, with France achieving +15.2 ± 3.8 points and Cameroon +13.8 ± 4.2 points (*p* = 0.28, independent *t*-test). Both improvements substantially exceeded the minimal clinically important difference (MCID) of 1.7 points by 8.9× and 8.1× respectively. Panel **(**B**)** shows spasticity reduction measured by the Modified Ashworth Scale (MAS), with France demonstrating −1.3 ± 0.4 point reduction and Cameroon −1.1 ± 0.5 points (*p* = 0.33). Error bars represent standard deviations. No statistically significant differences were observed between sites for either primary or secondary outcomes (all *p*-values >0.05), supporting the conclusion that family-mediated manual therapy produces equivalent clinical benefits across dramatically different resource environments. Within-group improvements were highly significant for both cohorts (*p* < 0.001) for both GMFM-66 and MAS outcomes. Effect sizes (Cohen's d) exceeded 1.8 for both groups, indicating very large treatment effects. These results demonstrate that the Cameroonian home-based model achieved comparable motor function improvements and spasticity reduction at one-eighth the cost (38€ vs. 320€ monthly) of the French institutional model, supporting scalability to resource-limited settings.

Subgroup analysis by GMFCS level revealed consistent gains across severity strata in both cohorts. Children at GMFCS level I achieved the largest absolute gains (France: +18.1 ± 2.9; Cameroon: +16.4 ± 3.1), followed by level II (France: +15.3 ± 3.4; Cameroon: +14.1 ± 4.0) and level III (France: +11.2 ± 2.8; Cameroon: +10.7 ± 3.9). Between-group differences remained non-significant across all GMFCS strata (all *p* > 0.05), confirming that the equivalence of outcomes between settings was not driven by a specific severity subgroup. The small sample size within each stratum limits the statistical power of these comparisons and warrants cautious interpretation. The improvement in GMFM-66 scores exceeded the minimal clinically important difference threshold of 1.7 points established in previous research (32), with average gains approximately 8–9 times this threshold value Figure 3.

Reduced spasticity as measured by the Modified Ashworth Scale was observed across all muscle groups assessed, with the most pronounced effects in plantar flexors and hip adductors. This reduction in muscle tone corresponded with improved functional performance, particularly in standing and transitional movements.

### 3.3 Exploratory cost comparison

Dramatic differences in resource utilization and costs were observed between settings (Table 4), highlighting the economic efficiency of the family-mediated approach in the low-resource context.

**Table 4:** Economic and accessibility indicators.

Indicator	France (*n* = 9)	Cameroon (*n* = 12)	Ratio
Intervention Delivery			
Professional therapy sessions/week	2	2	1:1
Session duration (minutes)	45–60	45–60	1:1
Home practice sessions/week (family-led)	10–14	10–14	1:1
Resource Utilization			
Professional therapy hours/month	18–24	18–24	1:1
Family practice hours/month	40–56	40–56	1:1
Total therapeutic contact hours/month	58–80	58–80	1:1
Economic Indicators			
Monthly program cost (€)	320 ± 45	38 ± 8	8.4:1
Professional fees	280	0*	—
Facility costs	35	0	—
Transportation	5	38	0.13:1
Cost per GMFM-66 point gained	21.1 €	2.8 €	7.5:1
Accessibility Indicators			
Setting	Institutional	Home-based	—
Distance to facility (km), mean	8.2 ± 3.4	0^†^	—
Transportation barriers	Minimal	Substantial^‡^	—
Waiting time for initial access (weeks)	2–4	0	—
Program Outcomes			
Completion rate (%)	90% (9/10)	86% (12/14)^§^	—
Adherence to home program (%)	92.3 ± 6.1	89.7 ± 7.8	—
Family satisfaction (0–10)	9.1 ± 0.6	9.4 ± 0.5	—

Economic and accessibility comparison highlighting dramatic cost-efficiency of the Cameroonian home-based model while maintaining equivalent clinical outcomes. Despite 8.4-fold lower total costs (primarily due to elimination of professional fees and facility expenses), the adapted approach achieved comparable motor improvements (+15.2 vs. +13.8 GMFM-66 points, *p* = 0.28) and similar program adherence (92% vs. 90%). The home-based model demonstrated superior cost-effectiveness (2.8€ vs. 21.1€ per GMFM-66 point gained). Transportation remained the primary cost barrier in Cameroon, accounting for 100% of program expenses, while representing only 1.6% of costs in France. High completion rates and family satisfaction across both settings support intervention feasibility and acceptability.

* Professional fees covered by public healthcare system in Cameroon. Home-based intervention eliminates facility travel. Several families traveled 15–28 km on difficult terrain for therapist home visits. Excludes 3 deaths from causes unrelated to intervention.

† Home-based intervention eliminates facility travel (distance = 0 km for Cameroonian participants).

‡ Several families traveled 15–28 km on difficult terrain for therapist home visits.

§ Completion rate excludes the 3 deaths from causes unrelated to the intervention (calculated from 14 initially enrolled Cameroonian participants).

This economic comparison is explicitly exploratory and does not constitute a formal cost-effectiveness analysis. The figures presented reflect direct material costs only and do not account for therapist time, opportunity costs of caregiver participation, transport costs, or indirect costs. A full health economic analysis incorporating these dimensions would require prospective costing methodology beyond the scope of this retrospective observational study. Regarding data collection for costing variables: in France, cost data were extracted retrospectively from institutional billing records and statutory physiotherapy reimbursement schedules (Nomenclature Générale des Actes Professionnels, NGAP). Professional fees (280€/month) reflect the standard tariff for twice-weekly 45–60 min sessions reimbursed by the Assurance Maladie; facility costs (35€/month) were estimated from institutional overhead allocation records. In Cameroon, cost data were collected prospectively through structured therapist expense logs recording transportation costs per home visit, which constituted the sole direct program expenditure (38€/month). Household income figures were self-reported by caregivers during baseline interviews. These figures capture direct material costs only; a comprehensive incremental cost-effectiveness analysis would require prospective costing including indirect costs and caregiver opportunity costs.

The cost differential was particularly striking, with the Cameroonian implementation requiring approximately one-eighth the financial resources while achieving comparable clinical outcomes. This efficiency was primarily achieved through task-shifting from professionals to trained family members and eliminating facility-based expenses.

Transportation represented the largest barrier to participation in Cameroon, accounting for nearly all program costs. Despite this challenge, families demonstrated remarkable commitment, with several traveling considerable distances (up to 28 km on difficult terrain) to ensure therapist access.

### 3.4 Qualitative outcomes: social impact and stigma reduction

Qualitative data were collected through semi-structured interviews conducted by the principal investigator with all Cameroonian caregivers at program completion. Interview themes included perceived changes in family dynamics, community perception of the child, caregiver burden, and program sustainability. Responses were analyzed using thematic analysis following Braun and Clarke's framework, with themes identified inductively from the data. This exploratory qualitative approach is recognized as appropriate for initial inquiry in resource-limited settings where more structured designs are not feasible. Semi-structured interviews with families revealed profound social impacts extending beyond motor improvements. Thematic analysis identified five major domains of social change:

1. Community perception: 11/12 Cameroonian families reported improved community attitudes toward their child, with reduced use of derogatory terminology and increased social acknowledgment.
2. Family dynamics: 7/12 families noted greater extended family involvement and support after witnessing functional improvements; 2/12 reported renewed contact with previously disengaged fathers.
3. Child participation: All families described increased participation in family activities and daily routines, with emphasis on mealtime integration and personal care independence.
4. Caregiver confidence: Mothers and grandmothers reported dramatically increased confidence in handling their children, reducing anxiety about causing harm through movement.
5. Future outlook: 9/12 Cameroonian families expressed new aspirations for their child's future, including school attendance and social integration, which they had previously considered impossible.

#### 3.4.1 Representative testimonials from participating families provide qualitative insight into these impacts

“Before, people called him a snake-child. Now he plays outside, and the neighbors see him differently since he can sit up by himself.” — Mother of 8-year-old boy, Bafoussam

“I was ashamed to take her out. When she walked to the gate with her walker, the whole neighborhood clapped. My sister-in-law who refused to touch her now helps with her exercises.” — Grandmother, Bamougoum

“The biggest change is that I'm not afraid anymore. I know how to hold him, how to help him move. Before I thought I would break him if I tried to stretch his legs.” — Mother, Foumbot

### 3.5 Six-month follow-up (Cameroon subset)

Limited follow-up data from Cameroon (8/12 participants) at six months post-intervention suggested maintenance of functional gains, with mean GMFM-66 scores showing slight additional improvement (+2.3 points beyond immediate post-intervention). Families reported continued adherence to modified home programs, with gradually declining frequency but sustained implementation of key techniques.

Four families had independently trained additional family members in basic techniques, creating a small-scale diffusion effect within their communities. Two children had begun attending school, representing a major shift in social inclusion previously considered unattainable.

## 4 Discussion

### 4.1 Interpretation of key findings

The comparable motor improvements observed across dramatically different rehabilitation contexts support our primary hypothesis that family-mediated manual therapy can achieve meaningful outcomes in resource-limited settings when appropriately contextualized. The magnitude of improvement in both cohorts (≈14 GMFM-66 points over 12 weeks) exceeds typical gains reported in many conventional therapy protocols (33–35) and aligns with intensive models employing much greater professional resources (36, 37).

This effectiveness likely stems from several interrelated factors. First, the intensive, repetitive nature of the intervention, with daily practice sessions guided by a trained family member, provides significantly greater therapeutic “dosage” than weekly professional sessions alone. This aligns with established principles of neuroplasticity, which emphasize the importance of repetition in driving functional neural reorganization (22, 23). Second, the home-based, contextually relevant implementation embeds therapeutic activities within meaningful daily routines, enhancing both motivation and functional carryover (38). Third, the sequential skill-building approach ensures mastery at each level before progression, promoting confidence and competence among family implementers (27).

Perhaps most notably, these functional improvements were achieved at dramatically different resource levels, with the Cameroonian implementation costing approximately one-eighth that of the French model while no statistically significant between-group differences. This efficiency challenges conventional assumptions about necessary resource intensity for effective pediatric rehabilitation and offers a promising model for addressing the massive rehabilitation gap in similar settings.

Beyond motor outcomes, the profound social impacts documented through qualitative analysis highlight the potential of this approach to address the complex social dimensions of childhood disability in stigmatizing contexts. By demonstrating visible functional improvements to community members, the intervention appears to disrupt prevailing cultural narratives about the immutability and supernatural origins of disability. This shift in perception, in turn, facilitates greater social inclusion and participation, addressing dimensions of disability that extend beyond physical impairment.

The mortality observed in the Cameroonian cohort, while not directly related to the intervention, underscores the significant health vulnerabilities faced by children with disabilities in resource-limited settings. These deaths from preventable causes (malaria, respiratory infections) highlight how disability intersects with other health challenges in contexts of poverty and limited healthcare access. This reality reinforces the importance of embedding rehabilitation within broader health system strengthening and social support initiatives.

### 4.2 Mechanisms of efficacy: five innovative concepts

Based on our integrated quantitative and qualitative findings, we propose five conceptual mechanisms that may explain the efficacy of this approach, particularly in resource-limited and culturally complex settings:

#### 4.2.1. Ecological plasticity

We define ecological plasticity as the enhanced capacity for neuromotor reorganization that occurs when rehabilitation is embedded within culturally relevant, daily-life contexts rather than artificial clinical environments. This concept extends traditional neuroplasticity principles by emphasizing the importance of meaningful environmental affordances in driving functional neural adaptation (24, 39). By situating therapeutic activities within the child's natural environment using familiar objects and routines, the Cogni-Famille approach may optimize the brain's inherent capacity to reorganize in response to task-specific demands (29). Ecological plasticity addresses both neurological and participation dimensions of the International Classification of Functioning, Disability and Health (ICF) framework (30).

##### 4.2.1.1 Empirical support

In our study, this ecological plasticity was evidenced by the comparable GMFM-66 improvements across dramatically different settings (France: +15.2 ± 3.8; Cameroon: +13.8 ± 4.2, *p* = 0.28). Particularly telling was the superior performance on transitional movements and functional tasks among Cameroonian children who practiced within their natural home environment using contextually relevant objects. For example, children who used water jugs for weight-bearing activities and local farming tools for grasp-release training showed greater improvement in these specific functional domains than those who used standardized clinical equipment. This suggests that the brain's plastic response is optimized when rehabilitation occurs within meaningful, contextually relevant environments rather than artificial clinical settings.

#### 4.2.2 Therapeutic motherhood

This novel concept describes the process through which mothers (or other primary caregivers) transition from passive recipients of professional advice to active agents of therapeutic change, simultaneously transforming their caregiver identity and social status. In contexts where mothers of disabled children face blame and marginalization, becoming skilled providers of effective therapy can restore dignity and purpose (28). The visible improvements in the child's function serve as public validation of maternal competence, potentially counteracting stigmatizing community narratives. This empowerment represents a crucial psychosocial dimension often neglected in conventional rehabilitation models focused exclusively on child outcomes.

##### 4.2.2.1 Empirical support

This transformation was clearly documented in our qualitative interviews, with 11/12 Cameroonian caregivers reporting increased confidence and social status. The testimonial “Before, people called him a snake-child. Now he plays outside, and the neighbors see him differently since he can sit up by himself” exemplifies how visible motor improvements validated maternal competence and challenged stigmatizing narratives. Notably, the two cases where previously disengaged fathers renewed contact after witnessing functional improvements further supports this concept, suggesting that therapeutic competence can restructure not only community perceptions but also family dynamics.

#### 4.2.3 Sequential micro-learning

This term describes our approach to skill transfer, wherein complex therapeutic techniques are broken down into small, manageable components that build sequentially upon each other, with new skills introduced only after mastery of prerequisite techniques. This approach, adapted from educational psychology principles (25), appears particularly effective in contexts where caregivers may have limited formal education or high anxiety about performing therapeutic procedures correctly. The gradual progression from simple to complex skills builds confidence while preventing cognitive overload, a frequent barrier to successful skill transfer in family training programs (26).

##### 4.2.3.1 Empirical support

The high program adherence observed (France: 92.3%; Cameroon: 89.7%) despite vastly different educational backgrounds suggests the effectiveness of this approach in building caregiver confidence. Particularly notable was the finding that Cameroonian grandmothers with no formal education achieved comparable technique proficiency to French caregivers with secondary education, as evidenced by similar reductions in Modified Ashworth Scale scores (France: −1.3 ± 0.4; Cameroon: −1.1 ± 0.5, *p* = 0.33). The testimonial “The biggest change is that I'm not afraid anymore. I know how to hold him, how to help him move” directly illustrates how sequential skill-building transformed perceived self-efficacy.

#### 4.2.4 Community anchoring

We observed an unexpected diffusion effect wherein successfully trained families became informal rehabilitation resources within their communities, sharing knowledge and demonstrating techniques to others with similar needs. This phenomenon, which we term community anchoring, extends the impact beyond enrolled participants and potentially builds sustainable local capacity. The visible functional improvements of participating children serve as powerful evidence that challenges fatalistic community attitudes about disability prognosis (40). This mechanism aligns with community-based rehabilitation principles while emerging organically rather than through formal program design (41).

##### 4.2.4.1 Empirical support

Our six-month follow-up data from Cameroon revealed that four families had independently trained additional family members in basic techniques, creating a small-scale diffusion effect within their communities. This organic knowledge transfer represents a promising pathway for sustainable capacity building in resource-limited settings. Furthermore, the reported reduction in derogatory terminology used by community members suggests that visible functional improvements served as powerful evidence challenging fatalistic community attitudes. The transition of two children into formal schooling by the six-month follow-up further demonstrates how rehabilitation success can anchor new community perceptions of disability.

#### 4.2.5 Functional dignity restoration

Perhaps most fundamentally, the combination of improved motor function and reduced social stigma appears to initiate a process we call functional dignity restoration. This concept encompasses the mutually reinforcing cycle wherein improved physical function enhances social acceptance, which in turn motivates greater participation and further functional development. The child's emerging capabilities redefine their social identity from that of a “cursed” or “snake” child to a person with legitimate social membership, albeit with differences requiring accommodation (42). This reconceptualization extends beyond the individual to transform family and community perceptions about the nature and meaning of disability itself.

##### 4.2.5.1 Empirical support:

The profound social impacts documented through qualitative analysis support this concept. All 12 Cameroonian families reported increased participation in family activities and daily routines, with 9/12 expressing new aspirations for their child's future, including school attendance and social integration, which they had previously considered impossible. The transformation from “hidden” to publicly acknowledged status represents the essence of functional dignity restoration. As one grandmother stated: “When she walked to the gate with her walker, the whole neighborhood clapped. My sister-in-law who refused to touch her now helps with her exercises.” This narrative encapsulates how improved function catalyzes social acceptance, which in turn motivates greater participation in a mutually reinforcing cycle.

### 4.3 Comparison with existing literature

Our findings contribute to the growing body of evidence supporting family-centered approaches in pediatric rehabilitation (43, 44), while specifically addressing the adaptation of such approaches to culturally complex, resource-limited settings. The magnitude of motor improvements observed aligns with those reported by Novak et al. (45) and Morgan et al. (46) in their reviews of intensive rehabilitation approaches, despite employing substantially fewer professional resources through the family-mediated model.

The socio-cultural impacts documented extend beyond those typically reported in rehabilitation literature, which has predominantly focused on functional outcomes rather than stigma reduction or social participation (47). Our findings more closely align with community-based rehabilitation research from other African contexts, where social inclusion represents a primary goal alongside functional improvement (48, 49).

The economic efficiency demonstrated compares favorably with other models designed for low-resource settings. Bennet et al. (50) reported that parent-implemented interventions in Bangladesh achieved significant developmental gains at approximately one-fifth the cost of center-based services, similar to our cost ratio (51). found that family-centered approaches in South Africa required fewer professional resources while achieving comparable outcomes to conventional therapy, consistent with our observations.

An important contextual asymmetry between cohorts deserves acknowledgement. All French children had prior access to institutional rehabilitation, while 75% of Cameroonian children had never received formal therapeutic intervention. This differential exposure could bias outcome comparisons in two opposing directions: prior rehabilitation experience in France may have accelerated protocol response, while therapeutic naïvety in Cameroon may have amplified responsiveness to any structured stimulation. The comparable GMFM-66 gains observed despite this asymmetry suggest that the Cogni-Famille protocol's active ingredients — intensive ecological repetition, family-mediated continuity, and culturally anchored motivation — are sufficiently robust to overcome baseline contextual disparities. This finding reinforces the potential of family-mediated approaches in African contexts where absence of prior rehabilitation access should be viewed as an opportunity rather than a contraindication.

However, our study adds several unique dimensions to this literature. First, the bi-continental comparative design provides direct evidence of effectiveness across dramatically different resource contexts using identical outcome measures. Second, the specific focus on manual therapy techniques, rather than general developmental stimulation, addresses the significant gap in specialized intervention approaches adapted for low-resource settings. Third, the detailed documentation of social impacts provides critical evidence regarding the potential for rehabilitation interventions to address stigma and exclusion, dimensions often neglected in functional outcome studies (12, 49–53).

### 4.4 Limitations

Several important limitations must be acknowledged. First, the retrospective design and non-randomized nature of the study introduce potential selection bias and limit causal inference. Second, the relatively small sample size limits statistical power for subgroup analyses and generalizability. Third, the lack of long-term follow-up data from the French cohort prevents comparison of maintenance effects across settings. Furthermore, outcome measurement was conducted only at program completion without standardized reassessment at predetermined intervals. The absence of formal follow-up at 6 and 12 months post-intervention limits conclusions regarding the durability of motor gains achieved. Future studies should incorporate structured reassessment at 6, 12, and 24 months to evaluate maintenance effects and potential functional regression over time. Fourth, the use of the GMFM-66 as the primary outcome measure, while validated and widely used, captures only gross motor function and may miss improvements in other developmental domains.

Additionally, contextual differences between sites extended beyond the intervention delivery model to include fundamental socioeconomic, cultural, and healthcare system factors that could not be fully controlled in analysis. The inability to blind assessors to participant group assignment introduces potential measurement bias, though standardized assessment protocols were rigorously followed to minimize this concern (42, 45, 54).

The mortality observed in the Cameroonian cohort represents a significant limitation, as these deaths may have introduced survivor bias in the results. Children who survived may have had different baseline characteristics or family support structures compared to those who died, potentially influencing outcomes. Future research should incorporate more robust health monitoring and potential preventive measures to address comorbidities in this vulnerable population (46–49, 55–58).

Finally, while qualitative findings provide rich insight into social impacts, the lack of standardized measures of participation, quality of life, or stigma experience limits quantitative comparison of these important outcomes. Future research should incorporate validated tools to assess these dimensions alongside motor function (12, 51, 60–64).

## 5 Conclusion

The Cogni-Famille Pediatric Adaptation demonstrates that family-mediated manual therapy can achieve meaningful motor improvements for children with cerebral palsy across dramatically different resource contexts, with particular promise for addressing the substantial rehabilitation gap in settings where specialized services are scarce or inaccessible. Beyond motor outcomes, this approach appears to initiate important social changes, disrupting stigmatizing narratives and promoting greater inclusion and participation.

The five innovative concepts proposed—ecological plasticity, therapeutic motherhood, sequential micro-learning, community anchoring, and functional dignity restoration—provide a theoretical framework for understanding the multidimensional impacts of this approach. These concepts extend beyond traditional rehabilitation paradigms to address the complex interplay between physical function, family dynamics, and social participation.

The mortality observed in the Cameroonian cohort underscores the urgent need for integrated approaches that address both rehabilitation needs and broader health vulnerabilities of children with disabilities in resource-limited settings. Future implementations should incorporate appropriate health monitoring and preventive measures alongside rehabilitation interventions.

Future research should prospectively evaluate this approach with larger samples, longer follow-up periods, and more comprehensive outcome measurement including standardized assessment of participation and quality of life. Additionally, implementation science approaches are needed to identify optimal strategies for scaling this model to reach the millions of children with CP in similar settings who currently lack access to effective rehabilitation services.

The Cameroonian implementation generated practical insights that informed the French adaptation. The observation that grandmothers serving as primary caregivers demonstrated particularly high training retention — attributable to stronger intergenerational motivation and availability — led to a reconceptualization of caregiver selection criteria. Additionally, the spontaneous emergence of peer-support networks among Cameroonian families prompted the introduction of structured group caregiver sessions in the French institutional setting. These cross-contextual learnings illustrate the bidirectional nature of translational rehabilitation research between high- and low-resource environments, challenging the assumption that knowledge transfer flows exclusively from high- to low-resource settings.

Ultimately, the Cogni-Famille Pediatric Adaptation demonstrates that when families are empowered with appropriate skills and support, rehabilitation can become not only a clinical intervention but a social movement, transforming not just how children move but how they and their families are perceived and included within their communities.

## Data Availability

The raw data supporting the conclusions of this article will be made available by the authors, without undue reservation, subject to compliance with institutional regulations of the Regional Hospital of Bafoussam (Institutional Certification N°43/DRSO/HRB/55/2023) and applicable data protection requirements.
